# Stable transgene expression and CRISPR-mediated knock-in system of a bacteria-derived antibiotic selection gene in the green alga *Ulva prolifera*

**DOI:** 10.1186/s12870-025-07411-y

**Published:** 2025-10-06

**Authors:** Zheng Qin, Whelver Surnido, Hiroyuki Mizuta, Toshiki Uji

**Affiliations:** 1 Laboratory of Aquaculture Genetics and Genomics, Division of Marine Life Science, Graduate School of Fisheries Sciences, Hokkaido University, Hakodate, 041-8611 Japan; 2 Laboratory of Aquaculture Genetics and Genomics, Division of Marine Life Science, Faculty of Fisheries Sciences, Hokkaido University, Hakodate, 041- 8611 Japan

**Keywords:** Modular plasmid, Stable transformation, CRISPR knock-in system

## Abstract

*Ulva prolifera* is a fast-growing green seaweed that has garnered considerable interest in both fundamental and applied research. Here, we established a molecular tool by employing a selectable marker gene that allowed the isolation of *U. prolifera* cells integrating exogenous DNA. We developed a modular plasmid for expressing exogenous genes in *U. prolifera* based on the bacterial antibiotic-resistance marker, aminoglycoside phosphotransferase gene (*aph7”*). Integration of *aph7”* in macroalgae can generate transformants resistant to hygromycin B. In addition, we characterized the promoter region of the small subunit of ribulose-1,5-bisphosphate carboxylase/oxygenase gene (pUpRbcS) to drive the expression of *aph7”*. The transcripts were consistently confirmed from antibiotic-selected transformants, stably retaining the exogenous gene in the succeeding generations. Subsequently, a CRISPR-based knock-in system was established, facilitating the integration of *aph7”* cassette in the endogenous selection gene encoding for adenine phosphoribosyltransferase (*UpAPT*). *APT* gene can serve as an endogenous marker in algae that exhibits a lethal phenotype under cultivation with 2-fluoroadenine. The resulting knock-in mutants could resist the co-selection of the antibiotic hygromycin B and 2-fluoroadenine. Our results advance *U. prolifera* as a genetic platform, enabling functional research to elucidate *Ulva* biology, and to bring forth biotechnological utilization of algal resources.

**Supplementary Information:**

The online version contains supplementary material available at 10.1186/s12870-025-07411-y.

## Introduction

Macroalgae are ecologically and economically important marine organisms [1–3]. The green macroalga *Ulva* attracts considerable interest in fundamental and applied research [4]. *Ulva prolifera* is widely distributed in tidal flats, seashores, and brackish estuaries, which oftentimes causes environmental problems [5, 6]. This species is a potential algal model, with a considerable number of morphological and physiological studies [7, 8], as well as investigations of its molecular processes [9, 10].

Developing transgenic individuals by a transformation system requires selecting those cells that contain the integrated exogenous DNA [11]. Selection marker genes facilitate the identification and isolation of the transformed cells, critical in plant transformations [12]. They confer gains of enzymatic activities in the transformants, such as resistance functions against lethal dosages of herbicides, antibiotics, or toxins [13]. The development of various selection markers enhances plant transformation efficiency, which is essential for plasmid-based genome editing.

CRISPR/Cas9 system, a breakthrough in precision genome editing, combined with efficient selection markers, offers promising applications in genetic studies. To date, endogenous toxin-based selection markers such as adenine phosphoribosyl transferase (*APT*) and peptidylprolyl isomerase (*FKB12*) have been applied in seaweeds [8, 14]. However, endogenous toxin-based selection markers that co-target other genes may lower editing efficiency than a single mutation in seaweeds [14]. Other selection markers are based on induced visual changes such as the disruption of *phytoene desaturase* (*PDS*) and *MYB* transcription factors, phenotypically affecting pigmentation and developmental activities [15]. These phenotype-based markers are commonly employed in genome editing in higher plants [16, 17], but not in seaweeds. To overcome these limitations, antibiotic-based pre-selection for improving genome editing has been proposed [18–20], and a subsequent co-targeting of endogenous visual selection markers. The double-stranded break (DSB) induced by CRISPR/Cas9 facilitates exogenous gene insertion [21]. In place of knockout mechanisms, knock-in of exogenous genes via homologous recombination (HR) causes genetic modifications at the DSB locus [22]. Hence, a CRISPR-based knock-in system in *U. prolifera* could bring about improvements in gene and protein engineering to advance the domestication of *Ulva* species.

The development of molecular tools is important for elucidating complex seaweed physiology towards improved traits in new varieties. While seaweed genomic resources are increasing [23, 24], few molecular tools are described yet, especially the selection marker for the stable transformation and expression of exogenous DNA. In algae, hygromycin B (HygB) is a commonly used antibiotic for transformant selection. In red seaweeds, the expression of *Streptomyces hygroscopicus* aminoglycoside phosphotransferase gene (*aph7″*) in *Pyropia yezoensis* generated a strain that could survive in a HygB-supplemented medium [25, 26].

Furthermore, one crucial component in successful transgene expression is the appropriate driving promoters. Several *Ulva*-specific promoters have been isolated, such as the promoter of *U. prolifera* actin 1 (pUpActin1), which was confirmed to significantly enhance the transient expression of the glucuronidase (*GUS*) gene in *U. prolifera* compared to employing exogenous promoters such as from the Cauliflower Mosaic Virus 35S (pCaMV35S) [27]. The promoter of the small subunit of ribulose-1,5-bisphosphate carboxylase/oxygenase (pRbcS) from *U. mutabilis* transient expressed the bleomycin resistance protein (BleR) [28, 29]. Here, we isolated the pUpRbcS as an endogenous promoter to drive the *aph7”* expression.

Among several delivery methods, polyethylene glycol (PEG)-mediated transformation effectively introduces plasmid DNA into the protoplasts in higher plants [30]. PEG mediation is also applicable to *Ulva*, particularly in the cell wall-free gametes [29, 31], as established in the genome editing system in *U. prolifera* [8]. While considerable efficiency was obtained, we aimed to improve PEG transformation. The optimized PEG-Ca^2+^ mediated transformation achieved higher efficiency in higher plants [32]. Hence, we compared the PEG-Ca^2+^- and PEG-mediated transformation in *U. prolifera*.

In this study, an *aph7”* cassette was constructed to present a stable nuclear transformation in *U. prolifera*, alongside the promoter validation and transformation methods. We unveiled the significance of utilizing an endogenous promoter for proper transgene expression. This tool facilitates transgenesis in *U. prolifer*a, allowing the exploration of functional genetics in green seaweeds.

## Experimental procedures

### Biological materials

The *Ulva prolifera* strain E21 was harvested from Shimato River, Shimato, Kochi by Masanori Hiraoka [6]. The original strain was cultivated in the laboratory and identified by IST (Accession no. AB298320). The strain utilized in the study was lab-cultured *Ulva prolifera* E21 from Kochi University, Japan. The haploid male gametophytes were cultured in filter-sterilized seawater enriched with a Provasoli solution [33]. The culture conditions were set at 20℃ with a 14 h:10 h L: D cycle under white, fluorescent tubes (100 µmol photons m^−2^s^−1^). The culture medium was renewed weekly. *Ulva* was grown and parthenogenetically propagated.

### Design of a modular cloning framework for facile construct assembly

The modular vector was assembled by the restriction cloning strategy with the expression vector pBI221. The *UpRbcS* sequence (ON050966.1) was obtained from the NCBI database and aligned to the publicly available whole genome sequence (GCA_023078555.1) to locate the pUpRbcS. The transcriptional regulation elements were predicted by the online analysis program PlantCARE (http://bioinformatics.psb.ugent.be/webtools/plantcare/html/) [34]. The transcription starting site was identified by the NNPP v2.2 program (BDGP: http://www.fruitfly.org/seq_tools/promoter.html) [35]. pUpRbcS was amplified by KOD Plus Neo DNA polymerase (TOYOBO, Osaka, Japan) with primers PstI-pUpRbcS-F1 and BamHI-pUpRbcS-R1 carrying the corresponding restriction sites PstI/BamHI. The PCR condition followed an initial denaturation for 2 min at 94℃; 30 cycles of 10 sec at 98℃, 30 sec at 60℃, and 1 min at 68℃. PCR amplicons were purified by agarose gel electrophoresis (FastGene™ Gel/PCR Extraction Kit, Nippon Genetics, Tokyo, Japan), digested, and cloned into pBI221 (TaKaRa Bio, Shiga, Japan) to generate a pUpRbcS-GUS. The *aph7”* gene and terminator were amplified from *P. yezoensis* expression vector pPyElf1-aph7” [26], then cloned into pUpRbcS-GUS, replacing the *GUS* region. The plasmids were transformed into *Escherichia coli* DH5α competent cells by heat shock, then spread to an ampicillin plate. The internal EcoRI restriction site in *aph7”* was mutated by KOD-Plus-Mutagenesis Kit (TOYOBO, Osaka, Japan). The final delivery plasmid was validated by sequencing and designated as pRa7”.

### Hygromycin resistance assay

To evaluate hygromycin resistance (HygR), the gametes were incubated in a plate containing half-strength PES medium with 0, 1, 2.5, 5, 6, 7, 8, 9, and 10 mg mL^−1^ hygromycin B (HygB) for 30 days. Two biological replicates were carried out independently.

### Plasmid transfection methods

The gametes were concentrated by phototactic migration and promptly resuspended in a low salinity buffer (1 M sorbitol, 10 mM Tris-HCl at pH 7.5, 10 mM CaCl_2_, and 10% PES medium) [29], at a Hemocytometer cell density of 1.0 × 10^4^ cells µL^−1^. To compare the efficiency of the *Ulva* transformation buffers (UTB), 100 µL of gamete suspensions were gently mixed with either 100 µL of UTB1 (60% PEG 4000; weight/volume) or UTB2 (10 mM of Tris-HCl pH 7.5, 10 mM of CaCl_2_, 40% PEG 4000) [8, 29]. About 3–5 µg of plasmid DNA was used for each transformation. Then, the gametes were washed with sterilized filtered seawater and centrifuged at 5000 g for 10 min to obtain the gamete pellet after 20 min incubation with the buffers. The gametes were resuspended with half-strength PES medium and allowed to settle in circular plates. The plates were incubated at 20℃ in the dark for 36–48 h before HygB treatment (10 mg mL^−1^ for 6–8 weeks). Afterward, the surviving thalli were transferred to HygB-free half-strength PES medium. Two experiments were carried out independently.

### Generation of knock-in mutants by CRISPR/Cas9 system

The pRa7” plasmid containing pUpRbcS-aph7”-tCrRbcS region served as the template for the insertion of DNA fragments PCR amplification. The primer set PstI-pUpRbcS-F1 and aph7KI-R was used to amplify the knocked in fragments by PCR. The amplification condition was aforementioned. The knocked in target site of *UpAPT* and the sgRNA were similar to a previous study [8]. Ribonucleoprotein complex (RNP) preparation was also carried out similarly, with a Cas9 protein (New England Biolabs, M0646T) and sgRNA synthesized in vitro (New England Biolabs, E3322S). Briefly, 100 µL of gamete suspension was gently mixed with 100 µL of UTB1. The DNA insert (1 µg) was co-delivered with the RNP. The resuspended gametes were incubated at 20℃ in the dark for 36–48 h before the co-selection with 10 µM 2-fluoroadenine (2-FA) and HygB. Two experiments were carried out independently with the co-construction of *apt* single mutants as the control.

### DNA extraction and PCR analyses

To verify *aph7”* integration, genomic DNA from HygR transformants and wild-type (WT) strains was extracted by Chelex method as modified from HwangBo et al. [36]. The same strains were harvested for RNA extraction using the TRIzol reagent (Thermo Fisher Scientific, Waltham, MA) according to the manufacturer’s instructions. The RNA sample was treated by DNase (Ambion) before cDNA synthesis. About 500 ng of RNA sample was reverse transcribed with PrimeScript™ II 1 st strand cDNA Synthesis Kit (TaKaRa Bio, Shiga, Japan). Genomic PCR and PCR with the cDNA template analyses were carried out using KOD FX NEO. The primer pair aph7”-F1 and aph7”-R2 was used to amplify the *aph7”* region. The primer set for pUpActin1 and *UpActin1* was selected as the PCR control [27].

### Confirmation of knock-in at the *UpAPT* locus

The DNA was extracted from surviving thalli. A nested PCR approach was adopted using genomic DNA as a template, with four primer combinations primers as shown in Fig. 4a. Firstly, UpAPT-286F/UpAPT-1148R amplified the *UpAPT* gene, encompassing the knock-in site. This amplicon was used as a template in the following PCRs with the *aph7”* primers nested to the knock-in region. Specific PCR amplicons from these primers included the induced replacement of *aph7”* and the putative mutation sites.

## Results

### Growth Inhibition assay under different hygromycin B concentrations

After 30 days of incubation with HygB, the growth of thalli cultivated under 8 mg mL^−1^ was suppressed, and complete lethality was recorded at 10 mg mL^−1^ (Fig.S1). We selected HygB concentrations of 8 and 10 mg mL^−1^ for transformant selection in the subsequent experiments.

### Characterization of endogenous pUpRbcS

The amplified promoter region of the *UpRbcS* gene is 978 bp upstream of the ATG start codon. The *cis*-elements and TATA box in the pUpRbcS were predicted, which are mainly located within 1 kbp upstream of the gene (Fig.S2). The putative TSS is located at 650 bp upstream of the start codon, while the TATA-box is 29 bp to 25 bp upstream of TSS. These regions were included for plasmid construction and promoter activity confirmation.

### Transgene expression with endogenous promoters

**Fig. 1:**
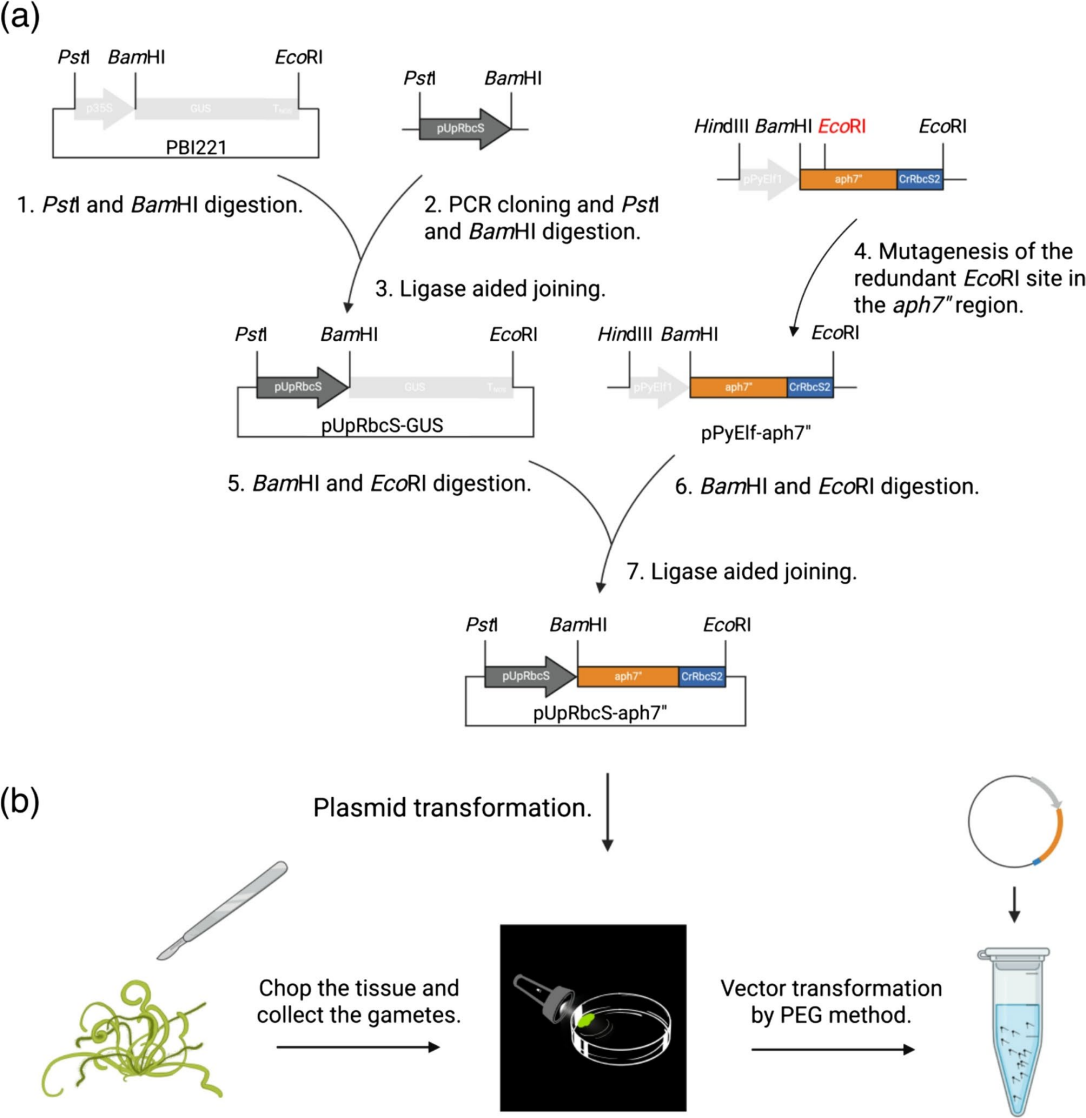
HygR Ra7” plasmid construction and algal transformation. (a) Schematic diagram of pRa7” plasmid construction and plasmid transformation. All insertion modules carry an ampicillin resistance cassette for bacterial selection. Only the Transfer-DNA region is shown. pUpRbcS: promoter of *UpRbcS* gene; *aph7*”: Hygromycin resistance *aminoglycoside phosphotransferase* gene; CrRbcS2: terminator of *RbcS2* from *Chlamydomonas reinhardtii*. (b) Induction of gametogenesis by fragmentation and gamete collection by positive phototaxis. The transformed gametes are haploid and developed parthenogenetically

**Fig. 2:**
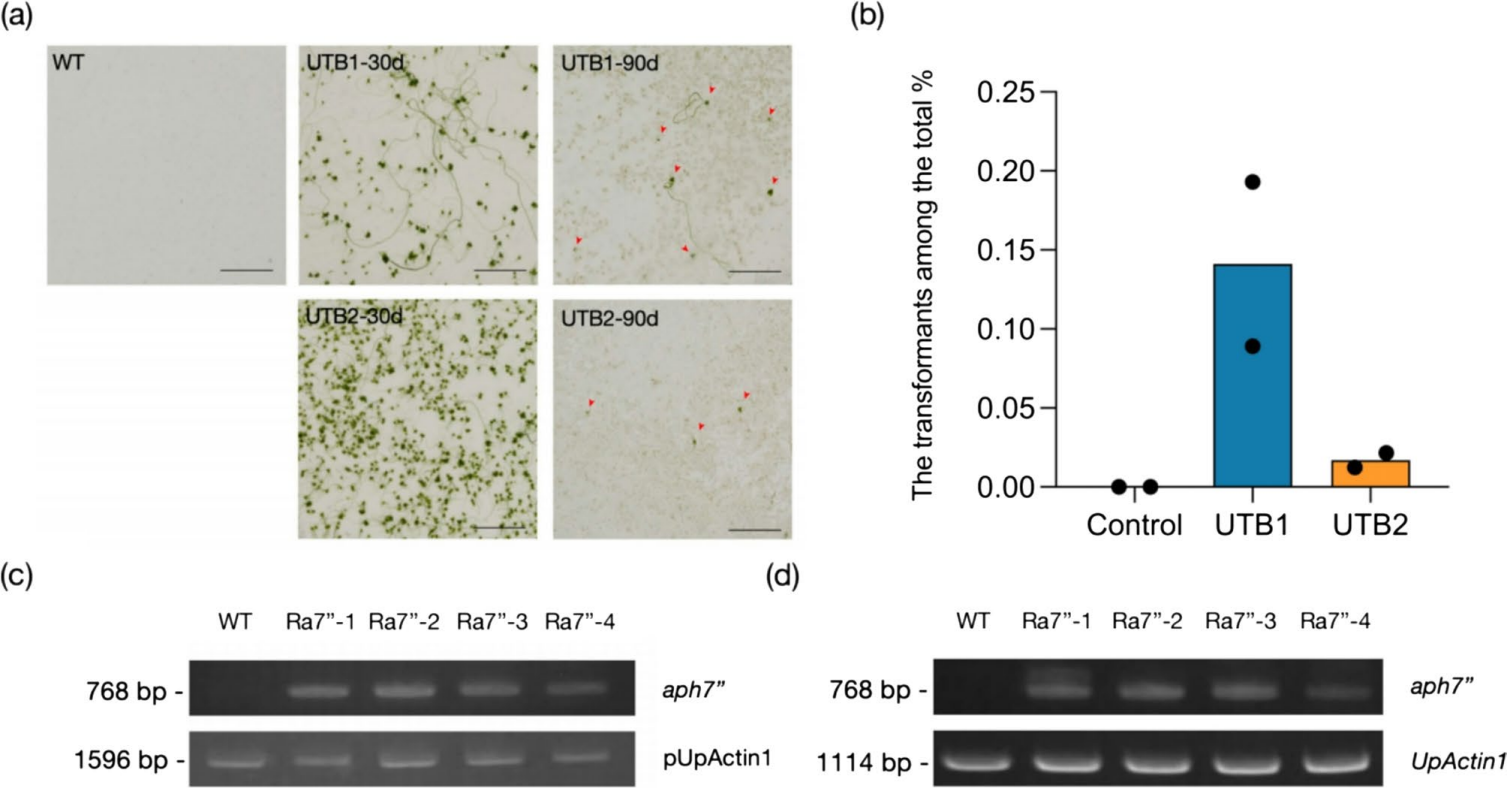
Ra7” transformant screening. (a) Transformants were obtained by two plasmid transformation approaches. The screening was carried out at 10 mg mL^−1^ HygB for 30 and 90 days.　The red arrow indicates the growing transformants. Scale bars: 500 μm (b) Survival rate of transformants for the transformation approaches at 10 mg mL^−1^ of hygromycin B. The mean survival rate was evaluated by two independent biological replicates comparing two transformation buffers. Expression of the exogenous *aph7”* gene in transformants and WT was detected by PCR with (c) genomic DNA and (d) cDNA as templates. pUpActin1 and *UpActin1* were used as PCR positive control

To verify the efficiency of pUpRbcS, we constructed the plasmid pRa7” (Fig. 1a). The purified pRa7” was transformed in the gametes via the PEG method with two transformation buffers, and the HygR gene *aph7”* as the selection marker. The attached gametes were haploid and developed parthenogenetically (Fig. 1b). UTB1 obtained 0.141% transformation efficiency, which was higher than that of UTB2 with 0.017% (Fig. 2a, b). We obtained more than 2000 colonies from the UTB1-exp1 delivered by UTB1 after 30 days of selection, in which the efficiency of transformation was shown as the highest (Fig. 2a, b). Under the 90-day selection, only Ra7” transformants could survive, but growth was inhibited at 10 mg mL^−1^ of HygB (Fig. 2a, b).

### Analysis of the bacterial *aph7* gene transcription

The expression of *aph7”* was confirmed by PCR with the genomic DNA and cDNA. Four transformants from each transformation were analyzed. Genomic PCR amplified the expected *aph7”* fragment from the transformants and not in WT (Fig. 2c). Similarly, the *aph7”* transcripts with the expected size were amplified only in the transformants (Fig. 2d). These validated that the exogenous *aph7”* gene had been successfully integrated and expressed in the four transformants. HygB selection also revealed a high proportion of HygR strains in the T1 generation (Fig.S3a, b). Moreover, we found that the T1 strains underwent reproduction during the HygB selection. This showed that *aph7”* could also be inherited into the T2 generation (Fig. S3a). The transgene was stably maintained in the genome of *U. prolifera* and its succeeding generations.

### Phenotype of CRISPR-mediated HygR cassette knock-in mutants

**Fig. 3:**
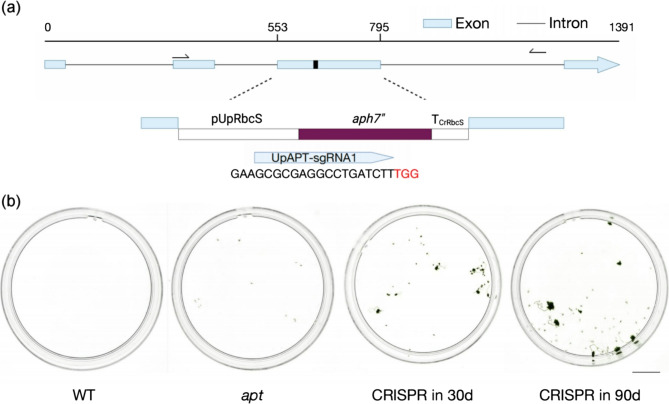
Generation and screening of RNP-based CRISPR in mutants. (a) Molecular map of the *UpAPT* exons and the locus of the integrated DNA fragment. The *aph7”* cassette was amplified from pRa7” plasmid. Bases with red indicate PAM sequence. (b) Phenotypic characteristics of wild types (WT), *apt* mutants, and the CRISPR in mutants. The mutants survived under 2-FA and HygB co-screening. CRISPR in 30 d, CRISPR in 90d: Co-screening for 30 and 90 days. Scale bar: 10 mm

Subsequently, employing HygR phenotypes, we developed a CRISPR-mediated knock-in (CRISPR in) system with a double-screening validation of CRISPR in. The HygR cassette was inserted in the *UpAPT* locus (Fig. 3a), and the resulting mutant exhibited resistance to co-selection of 2-FA and HygB for 90 days. Mutants were screened first with 2-FA. Then, we further selected *aph7”*-expressing strains with both 2-FA and HygB treatment for 90 days (Fig. 3b). All of the knock-in (KI) mutants survived under co-selection.

### Nested PCR screening and CRISPR-based knock-in confirmation at *UpAPT* locus

**Fig. 4:**
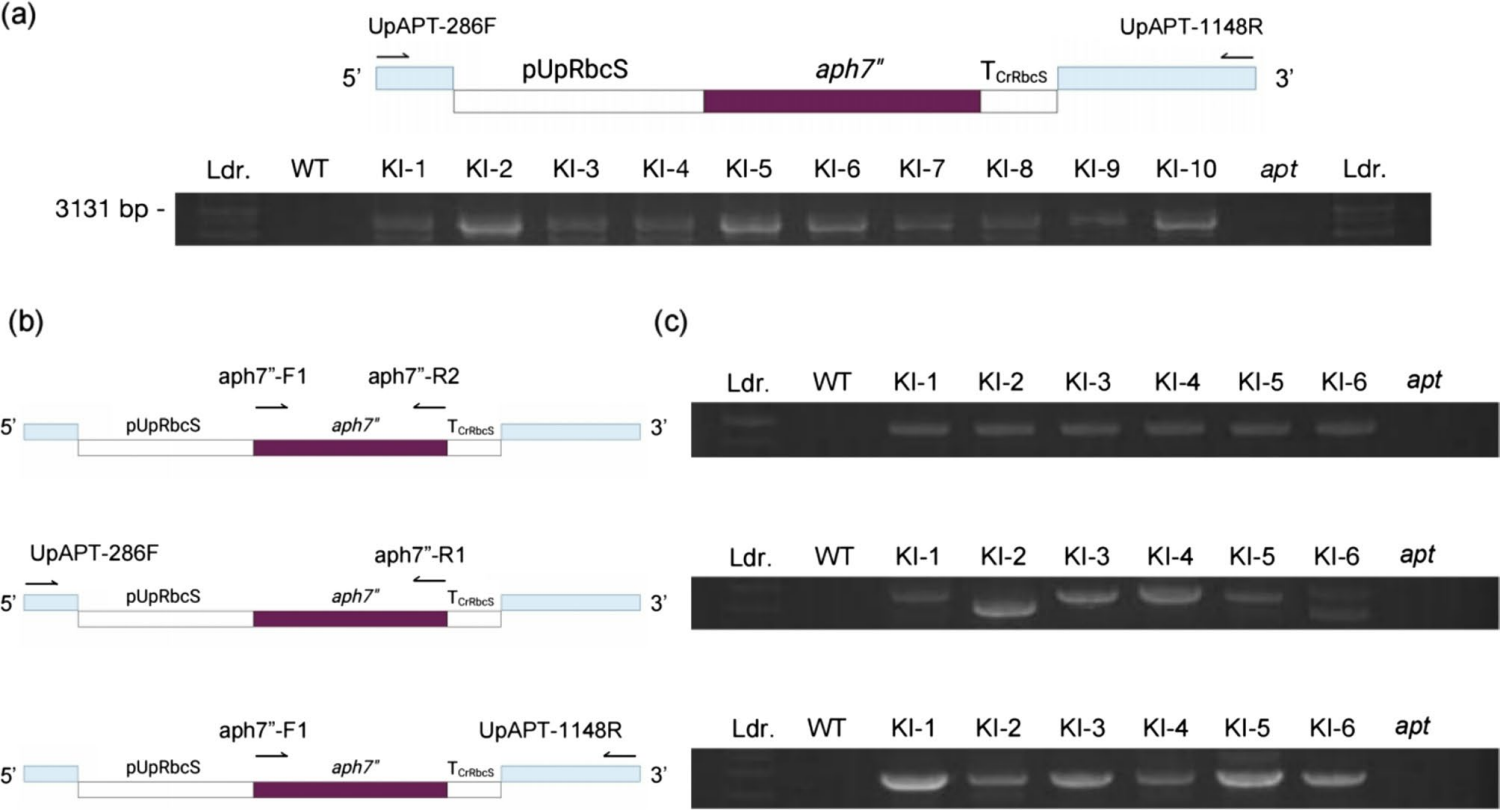
Mutant confirmation for gene deletion and knock-in by nested PCR. Primers target the 5’ upstream and 3’ downstream in the genomic region used for identifying the gene editing locus and the integration of HygR cassette. (a) PCR amplification of *UpAPT* locus. Amplicons of knock-in mutants showed a 3131 bp fragment that contained HygR cassette but not in WT. (b) Primers and the schematics of the coding DNA sequence of *aph7”* and (c) the nested PCR confirmation. Ldr: DNA ladder; WT: Wild type; KI: Knock-in mutants; *apt*: *apt* single mutant

The molecular nature of the *UpAPT* gene in KI mutants was analyzed by genomic PCR, amplifying the integrated HygR cassette in the Cas9 cutting site (Fig. 4a). The WT amplicon was smaller than that of the *apt* single mutants. The *UpAPT* amplified region was 862 bp, and 10 mutants showed amplification of around a 3000-bp fragment, indicating the size of the CRISPR in HygR cassette (Fig. 4a). Nested PCR with these amplicons as a template was carried out to cover the junction from the 5’/3’ end of *UpAPT* coding sequence and the HygR cassette (Fig. 4b, c). There was no amplification in the negative controls, i.e., WT and *apt* mutants. On the other hand, the target amplicons were confirmed in the KI mutants, suggesting a successful integration of the HygR cassette in the *UpAPT* locus by a CRISPR in method.

**Fig. 5:**
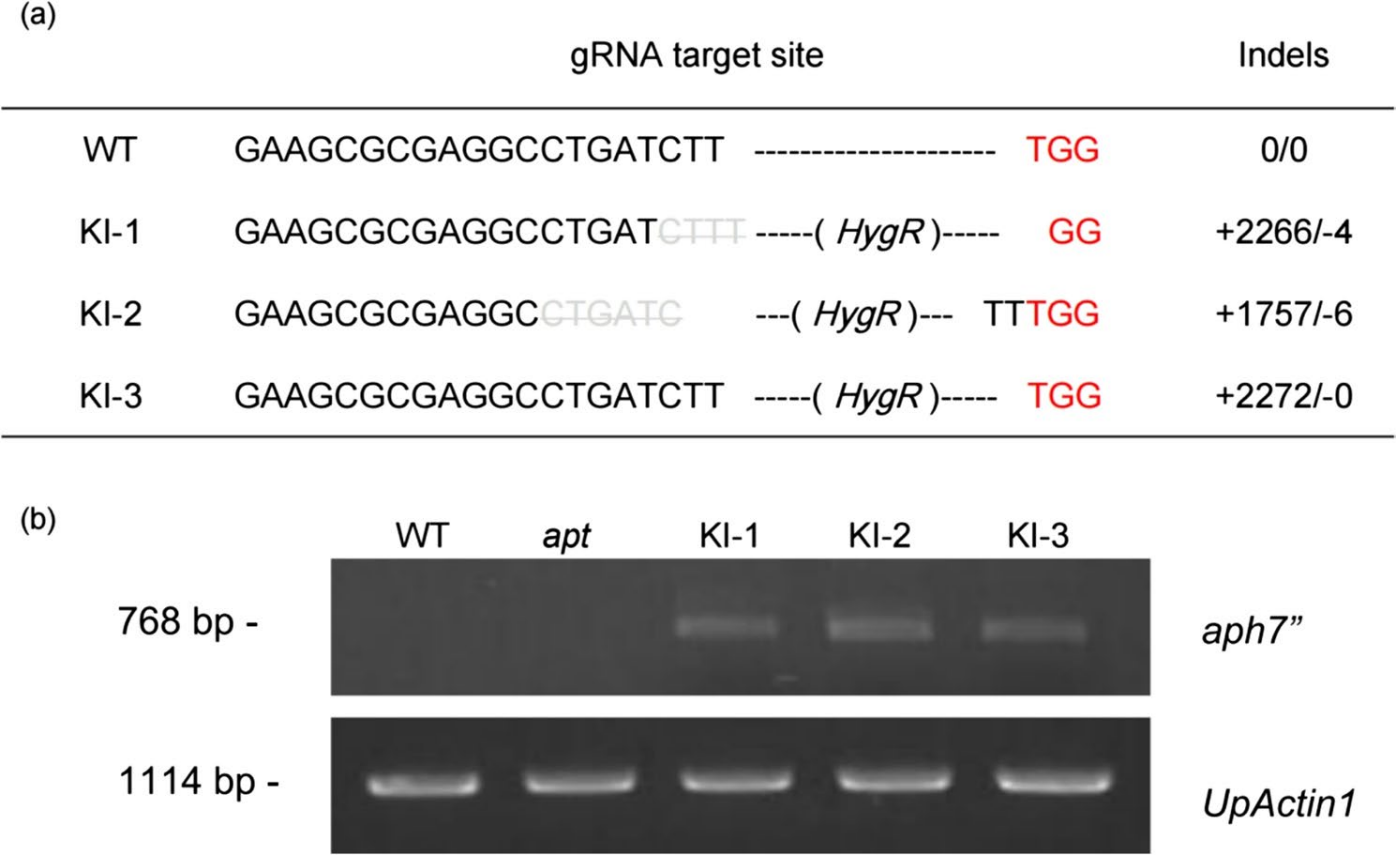
Validation of KI mutants by sequencing and cDNA-PCR. (a) Sequence comparison of WT and KI mutants. (b) cDNA-PCR analyses of *aph7”* transcripts in WT, *apt* single mutant, and KI mutants

Then, the *UpAPT* amplicons from the three KI mutants were sequenced. The amplicons from KI-1 and KI-2 contained base deletions at the knock-in target site, which could lead to frameshift mutations upon *UpAPT* translation. The HygR cassette in both KI-1 and KI-2 showed incomplete integration with losses in the far upstream part of pUpRbcS (Fig. 5a). On the other hand, KI-3 integrated the full-length HygR cassette at the target region without causing base deletions in *UpAPT* (Fig. 5a). Despite the integration of different promoter lengths, PCR comparison of the transcripts of KI-1, KI-2, and KI-3 showed amplification of *aph7”* (Fig. 5b). The deletion of the far upstream region of pUpRbcS did not affect the *aph7”* transcription. We confirmed the successful integration of the HygR cassette, resulting in the gain-of-function in HygR *aph7”* and loss-of-function of the *UpAPT*, as the resulting phenotypes.

## Discussion

With the rapid proliferation and completion of genomic resources [9], *U. prolifera* has become a model organism for green macroalgae. Prior to this study, only the BleR cassette was shown for stable transgene expression in *Ulva*, enabling transformant selection by phleomycin [4, 29]. The expression vector pBI221 includes multiple cloning sites and a reporter system, facilitating the construction of larger expression vectors capable of accommodating more modules with interchangeable markers [37]. We replaced the transient expression reporter *GUS* from the pBI221 vector with the HygR gene *aph7”*, driven by an endogenous *UpRbcS* promoter. Upstream of the *UpRbcS* gene, the typical promoter motifs such as TATA box and TSS were located far from the start codon, as also reported by Oertel et al. [29], which needed to be included in the engineered promoter for the successful expression *aph7”* in *U. prolifera*. This generated efficient transgenic *Ulva* lines that expressed a bacterial antibiotic resistance gene. This finding enriches the molecular toolkit for green seaweeds and complements macroalgal functional genetics for synthetic biology.

The efficiency of PEG-mediated transformation generated more than a hundred transformants in a single experiment. To evaluate the suitability of different transformation buffers for plasmid delivery, we compared PEG (UTB1) and PEG-Ca^2+^ (UTB2) transformation with the pRa7” plasmid. Although the CaCl_2_ in UTB2 influences the permeability of the cell membrane and is commonly used in protoplast transformations [38, 39], we found that the concentration of PEG 4000 mainly affected the transformation efficiency, regardless of Ca^2+^. The optimized conditions for transformation in *Ulva* gametes attained higher efficiency with 60% PEG as the transformation buffer.

*U. prolifera* exhibited moderate tolerance to HygB, and complete lethality to gametes and thalli was confirmed at 10 mg mL^−1^. In *U. mutabilis*, the selection efficiency with phleomycin was at 50 µg mL^−1^ [4, 29]. All *U. prolifera* WT thalli were susceptible to HygB, and no spontaneous mutants were observed in our laboratory cultivation. Although the *aph7”* gene is rich in G/C bases at 70.94% [40], *Ulva* species have a wide spectrum of G/C rich codons [29], which may adapt to the codon usage of high G/C transgenes such as *aph7”.*

To date, RNP-based genome editing in *Ulva* has been attained, targeting endogenous genes such as *APT* [8]. But reliance on endogenous genes as selective markers caused a noticeable transcriptomic change between the mutant and WT [41]. With the developed vector-based genome editing tool, gene mutation is independent of an endogenous selection marker, allowing the physiological comparison of the WT and mutant lines [15, 42]. Employing *aph7*”-based selection is one of the many available exogenous markers, which we aim to test similarly in future studies.

The ability to knock in exogenous DNA specifically and precisely into seaweed genomes is a critical step in fundamental seaweed biology towards precision breeding. Although the CRISPR/Cas9-mediated knock-in system has been described in *U. prolifera* by knocking in an *EGFP* gene [43], none has been reported for medium-based selectable transformation. In this study, a CRISPR/Cas9 knock-in of *aph7”* generated HygR mutants. Moreover, we applied rapid mutant pre-screening by nested PCR in the target locus. To further validate the candidate KI mutants pre-screened with nested PCR, the amplicons were analyzed. Targeting the *UpAPT* gene, we integrated a 2.2 kbp exogenous DNA fragment into the genome of *U. prolifera*. This generated the KI mutants that could survive the co-screening with HygB and 2-FA. Sequencing the mutation target of the KI-2 strain revealed a 500 bp loss in the far upstream of the promoter region of HygR cassette. The predicted *cis*-elements of pUpRbcS showed that the core promoter region is located around 500 bp upstream of the start codon (Fig. S2). Hence, the deletion of the far upstream region did not affect the function of *aph7”.* However, the mechanism of the losses of the HygR cassette remains unknown and will be explicated to improve the precision of our tool for integrating larger DNA fragments. Chromosomal insertion of large constructs of desired agronomic traits and metabolic pathways is a promising application in plant breeding [44]. In the future, we aim to develop *Ulva* as a molecular model for tagging endogenous genes, insertion of regulatory elements, and production of industrial compounds, advancing algal utilization in the field of molecular breeding and synthetic biology.

In summary, our research generated transgenic lines that stably expressed the HygR *aph7”* gene, validating its efficiency as a selection marker for stable nuclear transformation in *U. prolifera.* This result enriched the molecular toolkit of *Ulva*, enabling the establishment of a CRISPR in system and marking its importance in algal bioengineering.

## Supplementary Information

Supplementary Material 1.

Supplementary Material 2.

Supplementary Material 3.

## Data Availability

Data is provided within the manuscript or supplementary information files.
