# Supplementary material for: Stable transgene expression and CRISPR-mediated knock-in system of a bacteria-derived antibiotic selection gene in the green alga Ulva prolifera
Source: BMC Plant Biol. 2025 Oct 6;25:1323. doi: 10.1186/s12870-025-07411-y (PMC12502233; doi:10.1186/s12870-025-07411-y)
Supplement: Supplementary file 1 — Supplementary Material 1. [file 12870_2025_7411_MOESM1_ESM.docx]

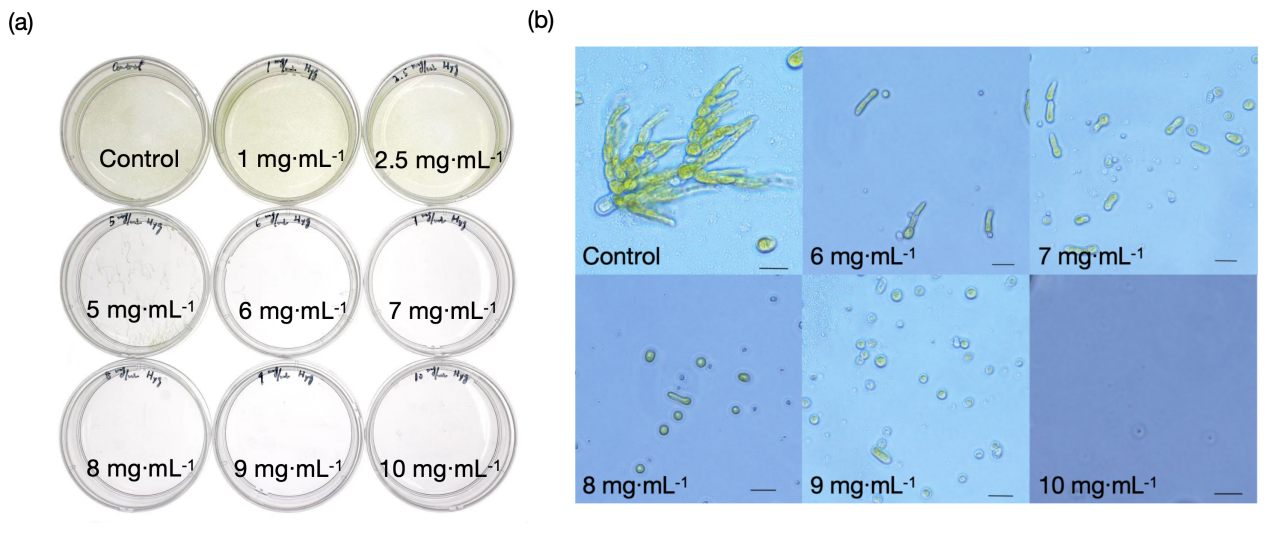


**Supplementary Figure 1.** Growth of *U. prolifera* under different HygB concentrations. Scale bar: 20 μm.


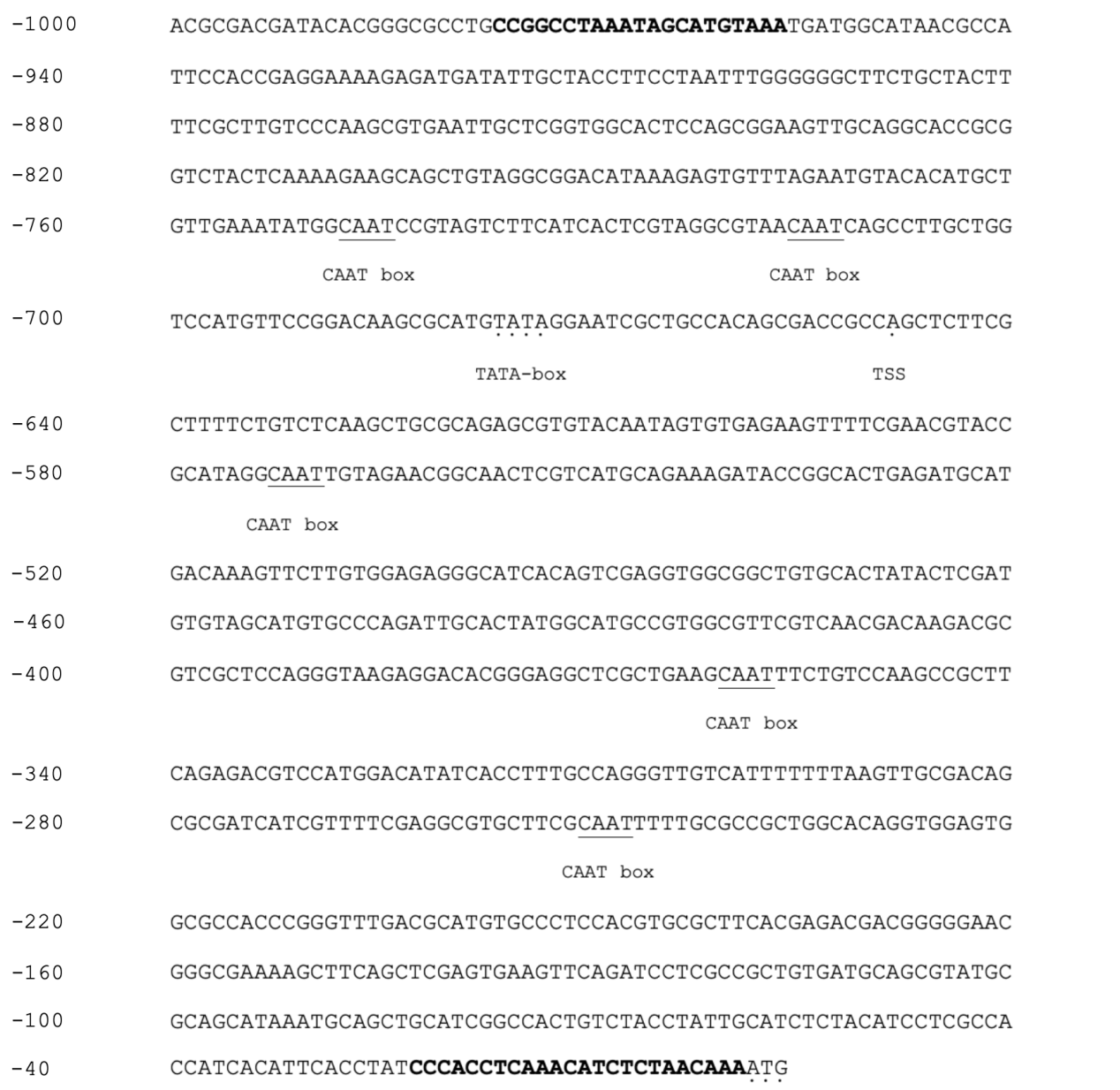


**Supplementary Figure 2**. Nucleotide sequence of the core pUpRbcS region and the predicted *cis*-elements. The numbers on the left start from ATG (+3). The bold nucleotides indicate the primers for core pUpRbcS amplification. The predicted common *cis*-acting CAAT box is underlined. The predicted TATA-box, transcription start site (TSS), and translational start codon (ATG) are indicated by dots below the sequence.


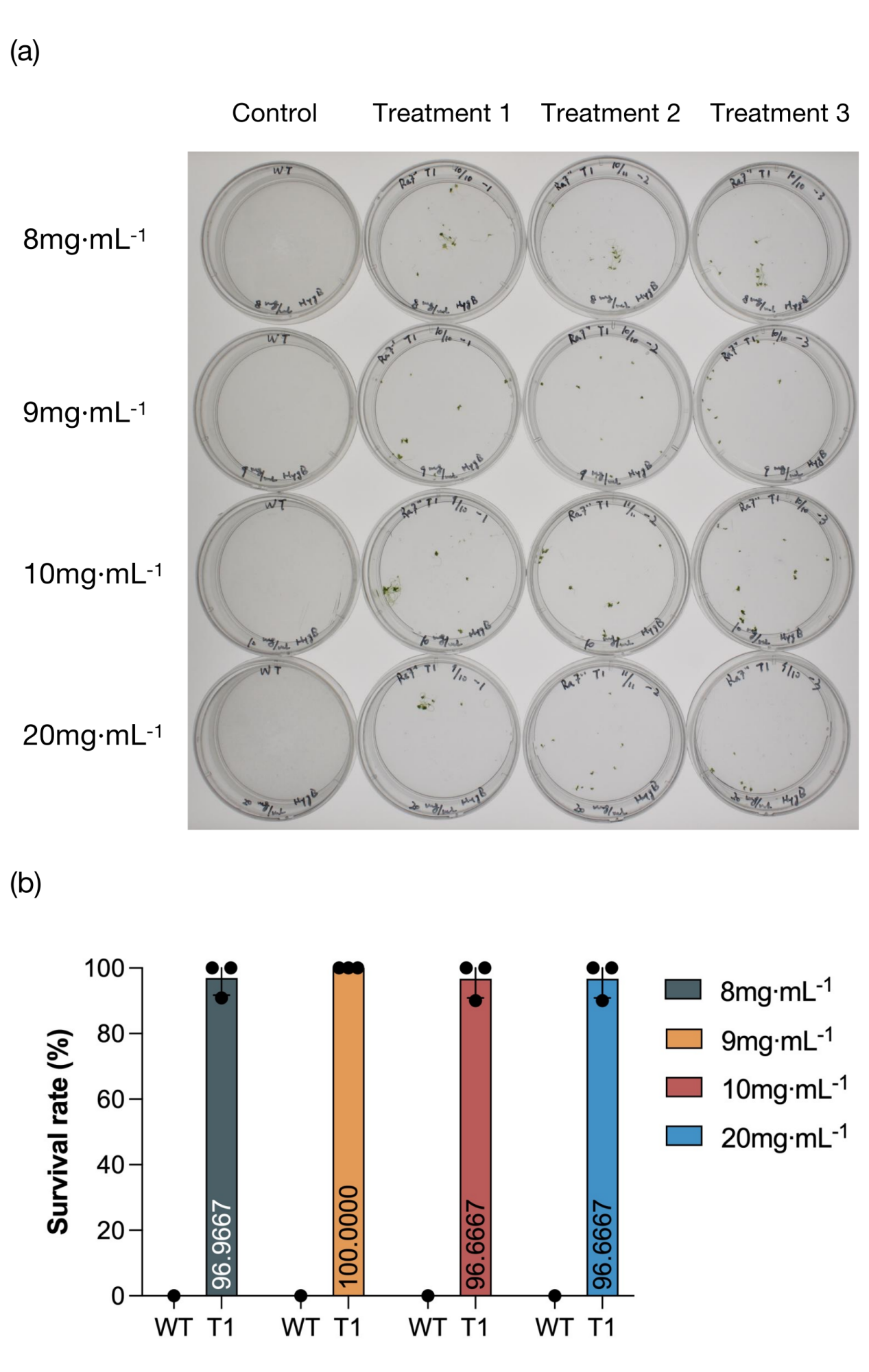


**Supplementary Figure 3.** (a) HygB selection of the first generation of Ra7” transformants (T1) and wild type (WT). Reproduction of T1 strain was observed during selections of less than or equal to 10 mg mL^-1^ HygB concentrations. This shows that the exogenous gene *aph7”* was inherited into the second generation (T2). (b) Survival rate during HygB treatment of Ra7”

T1. Error bars represent the standard deviations of the survival rate of three biological replicates. Data are presented as mean value ± S
