# Supplementary material for: Stable transgene expression and CRISPR-mediated knock-in system of a bacteria-derived antibiotic selection gene in the green alga Ulva prolifera
Source: BMC Plant Biol. 2025 Oct 6;25:1323. doi: 10.1186/s12870-025-07411-y (PMC12502233; doi:10.1186/s12870-025-07411-y)
Supplement: Supplementary file 3 — Supplementary Material 3. [file 12870_2025_7411_MOESM3_ESM.pdf]

Point-by-point response

3. We note that some of the gel/blot images in figure 2, figure 4 and figure 5 are heavily cropped. We ask that as full as possible length gels and blots are included in the Supplementary Information file. These images should be the original, unprocessed versions. We ask that you include all these images in one document, ensure that figure legends are added and that samples are labelled throughout.

genomic PCR of pRa7” transformants

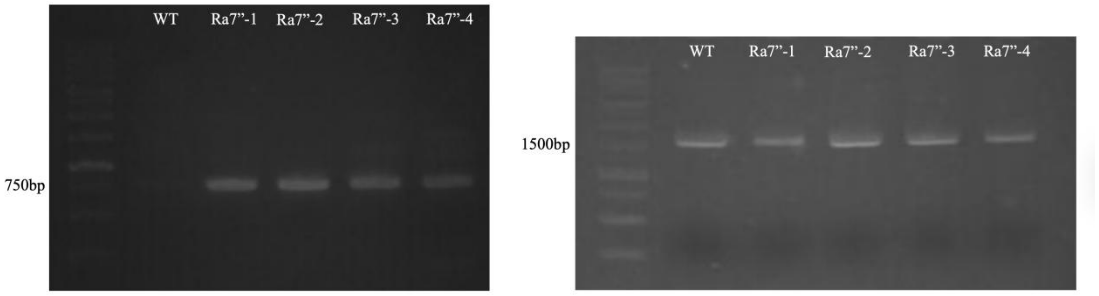

aph7” | pUpActin1 as control

Target sequence: 768 bp | Target sequence: 1596 bp

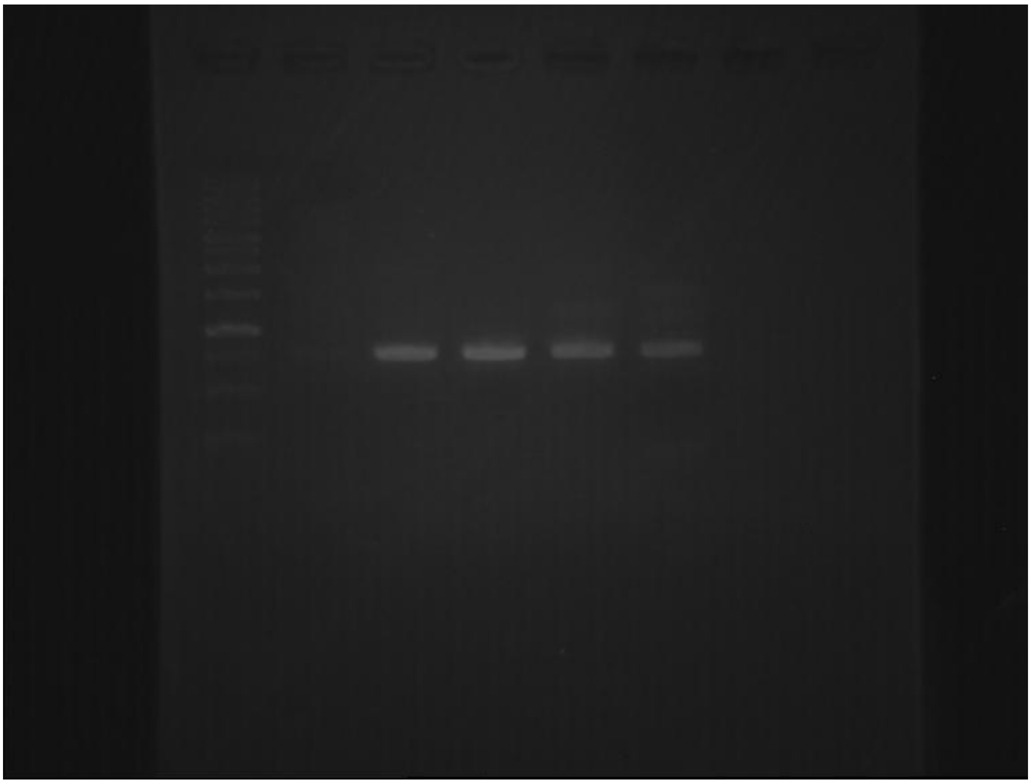

Fig.2c Left original figure

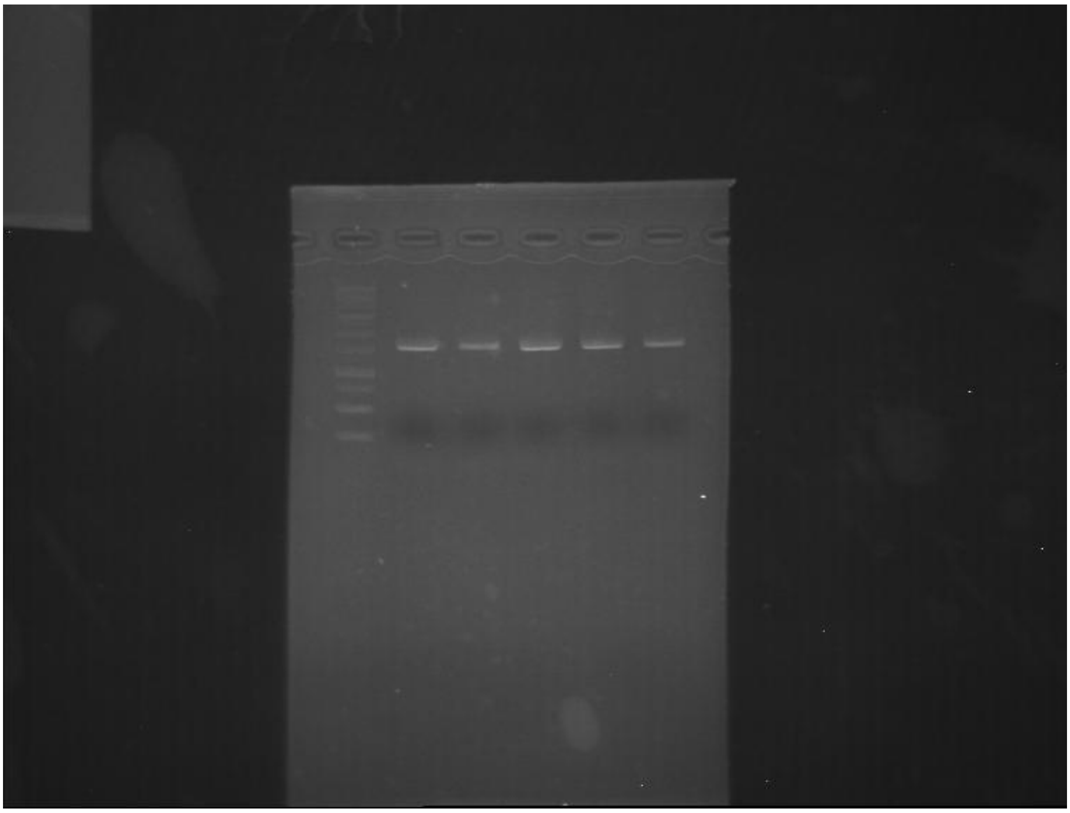

Fig.2c Right original figure

Fig.2(c).Expression of the exogenous *aph7”* gene in HygR transformants and WT was detected by PCR with genomic DNA. pUpActin1 and *UpActin1* were used as PCR positive control.

cDNA-PCR of pRa7" transformants

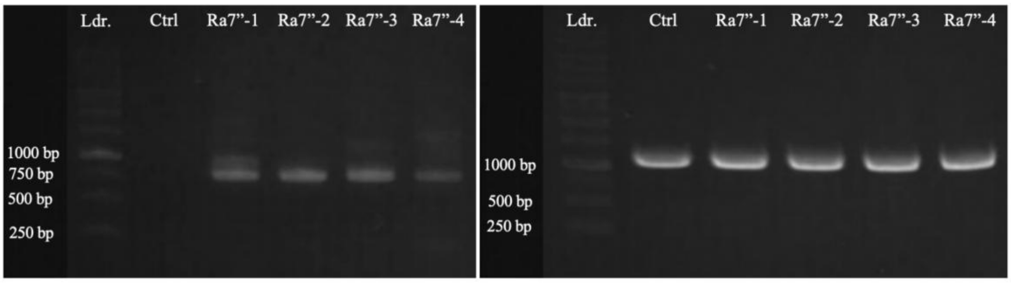

aph7" — Target sequence: 768 bp

UpActin1 as control — Target sequence: 1114 bp

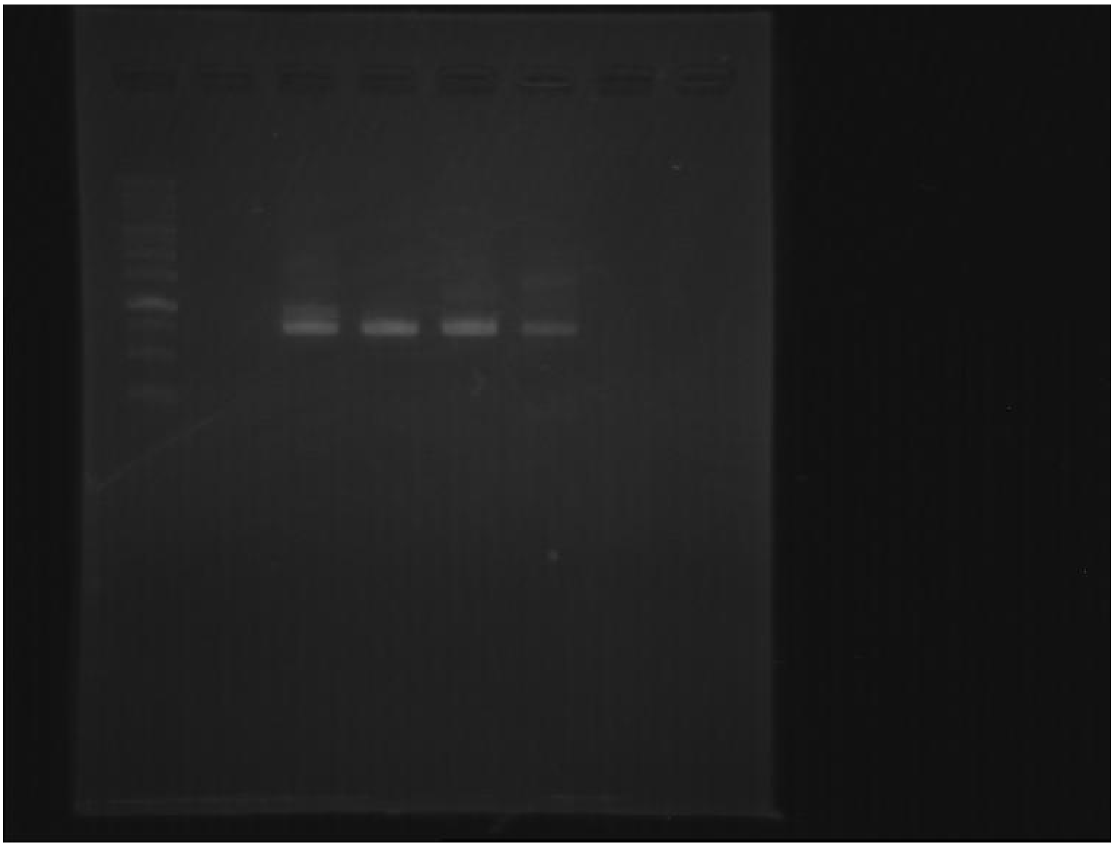

Fig.2d Left original figure

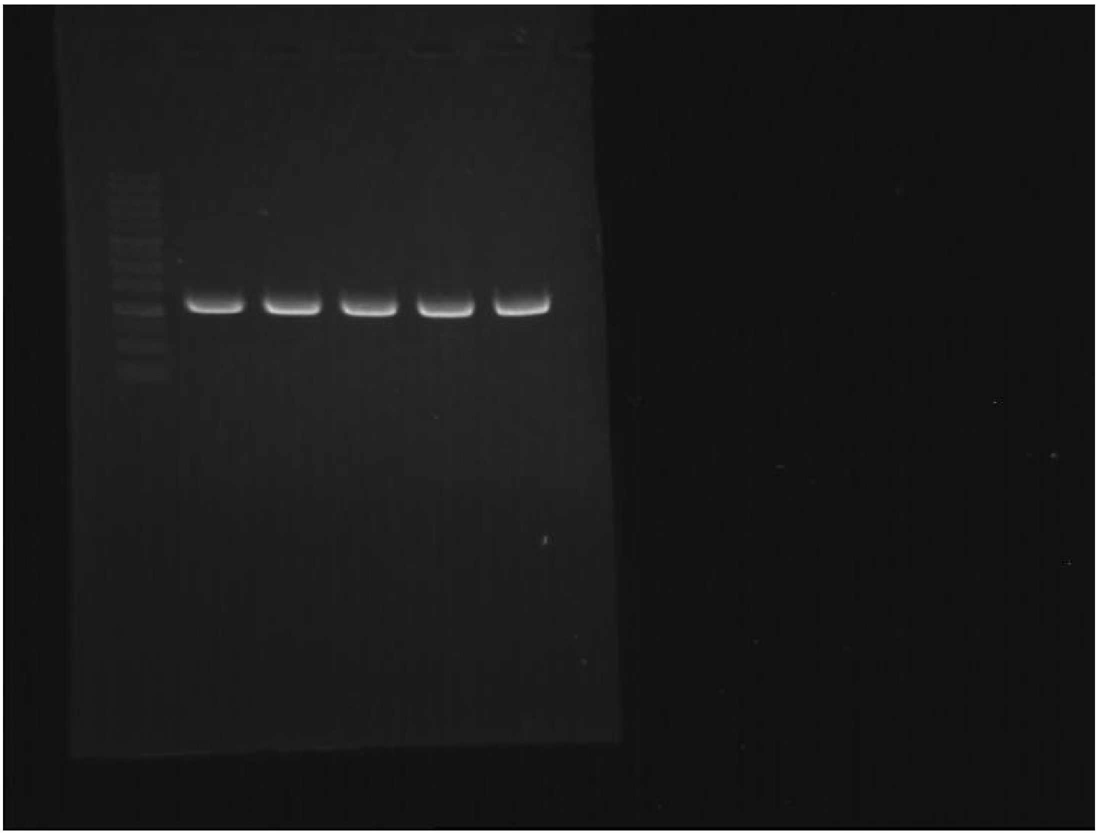

Fig.2d Right original figure

Fig.2(d). Expression of the exogenous *aph7”* gene in HygR transformants and WT was detected by PCR with cDNA. pUpActin1 and *UpActin1* were used as PCR positive control.

PCR confirmation of CRISPR- based *aph7"* knock-in mutants

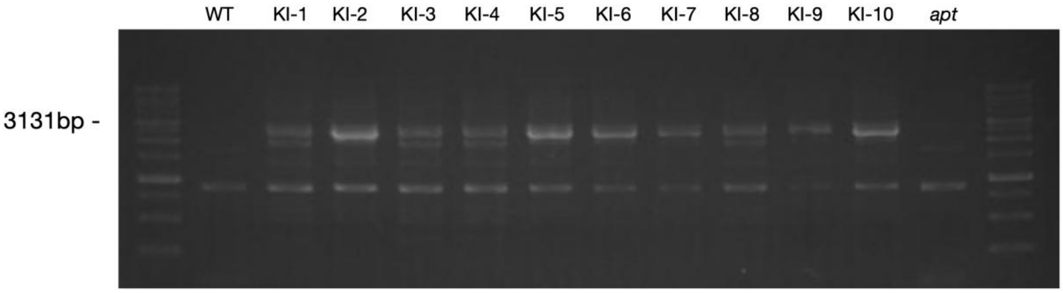

Knock-in fragment: 3131bp

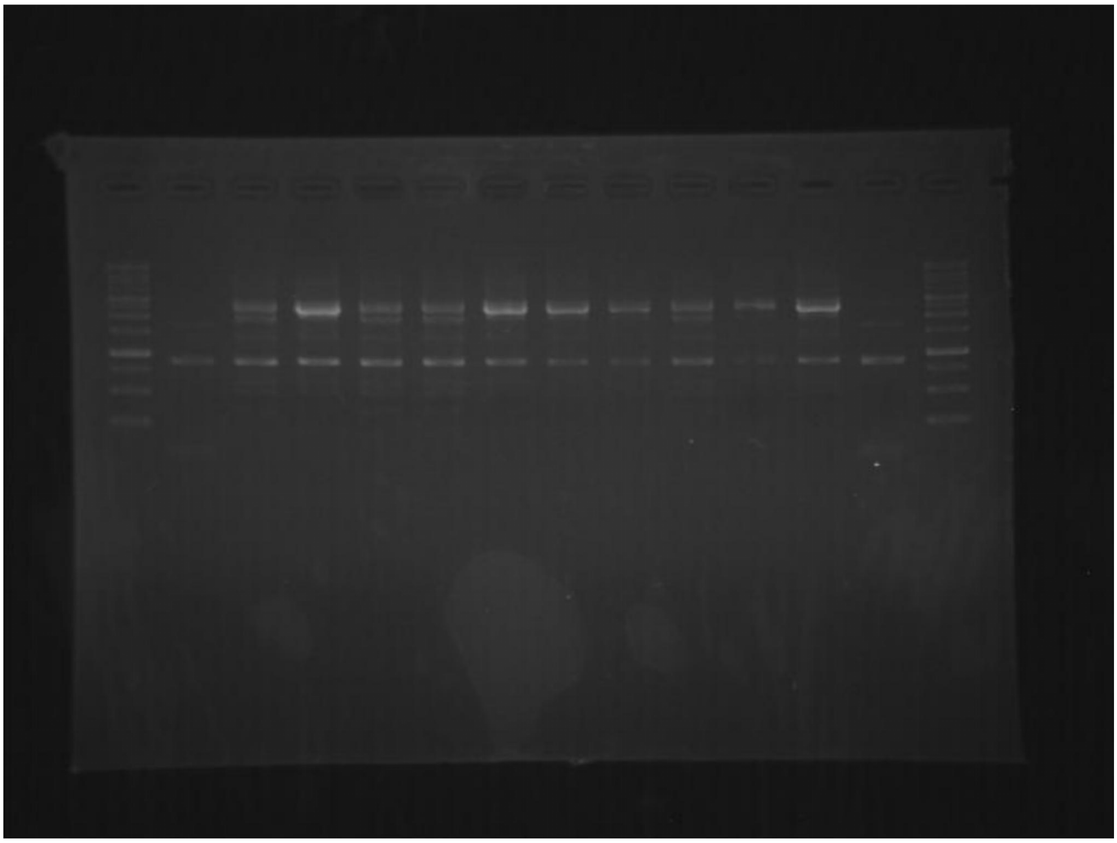

Fig.4(a). PCR amplification of *UpAPT* locus. Amplicons of knock-in mutants showed a 3131 bp fragment that contained HygR cassette but not in WT.

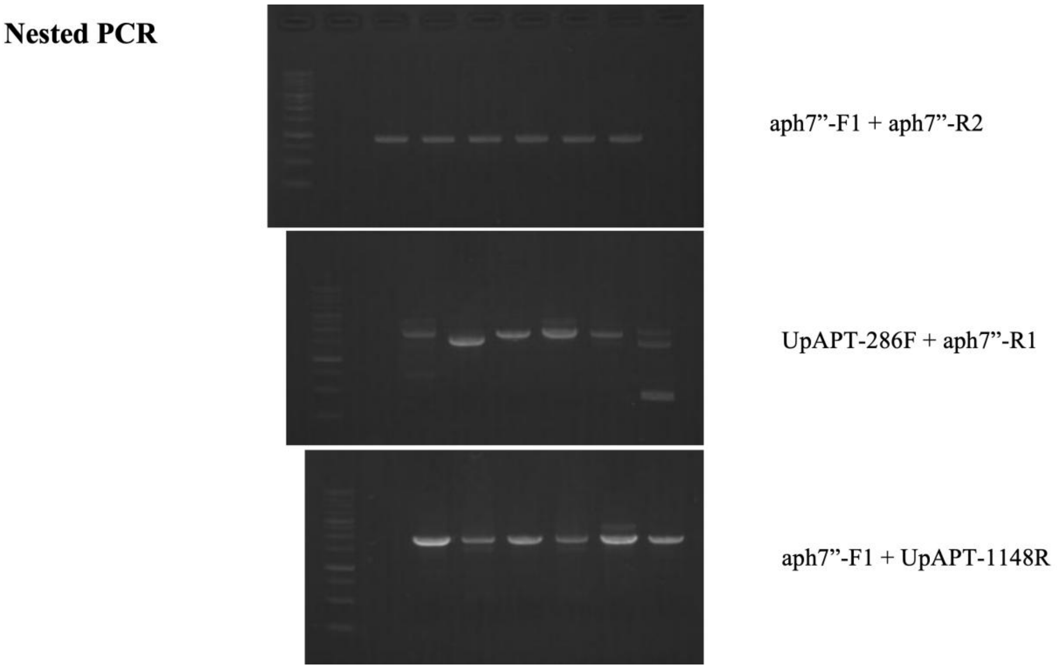

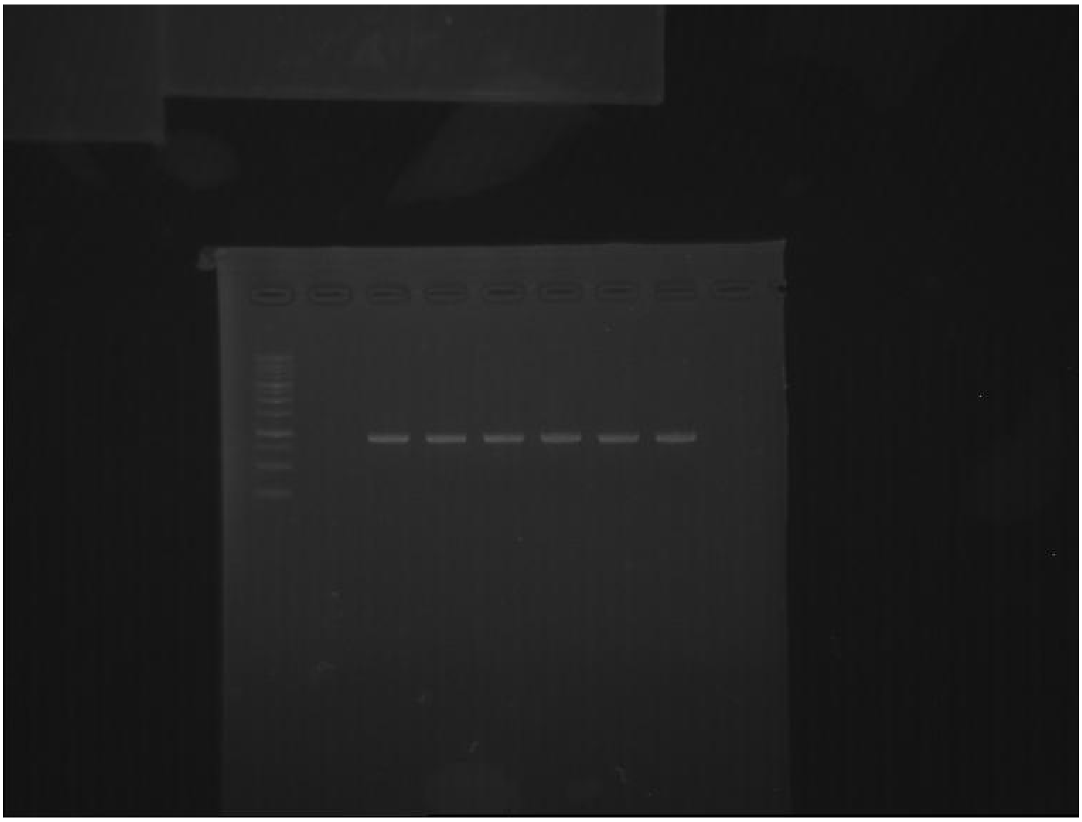

Fig.4c Top original figure

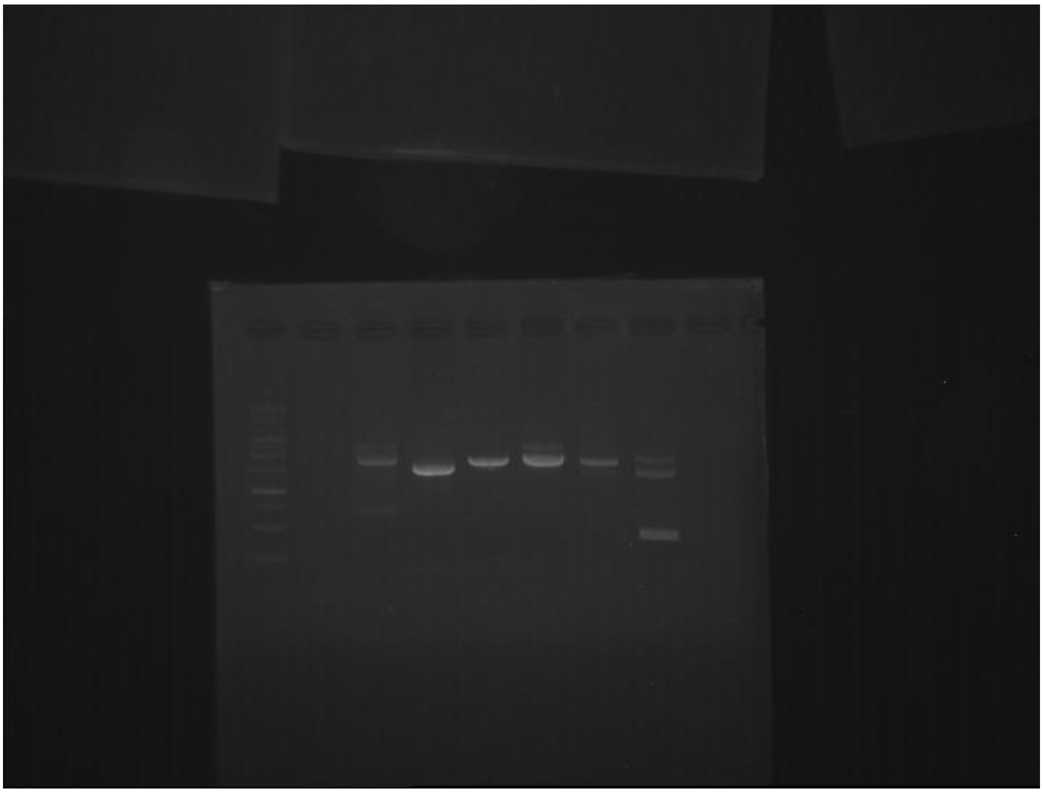

Fig.4c Middle original figure

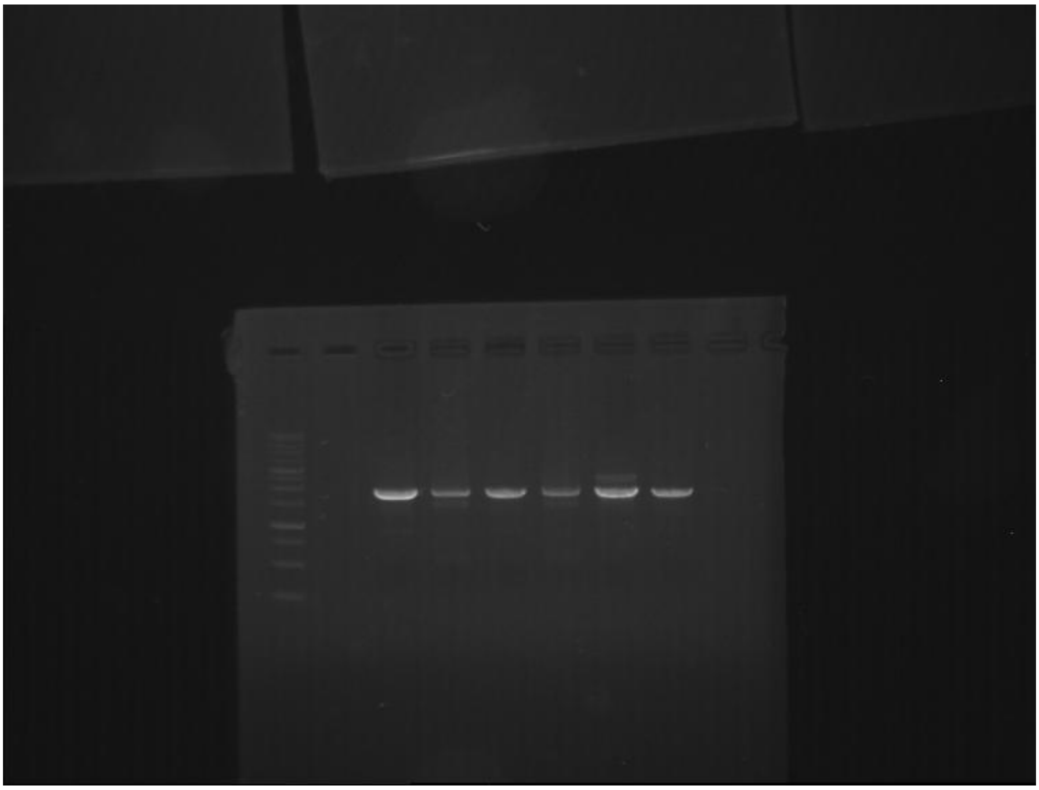

Fig.4c Bottom original figure

Fig.4(c). Nested PCR confirmation with different primer pairs. Ldr: DNA Ladder; WT: Wild type; KI: Knock-in mutants; *apt*: *apt* single mutant.

PCR analyses of *aph7”* transcripts

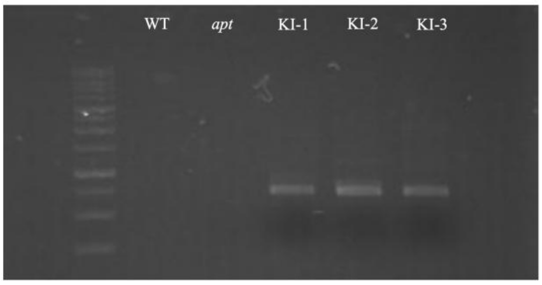

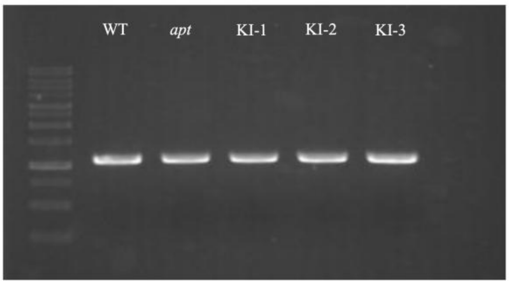

aph7”

Target sequence: 768 bp

UpActin1 as control

Target sequence: 1114 bp

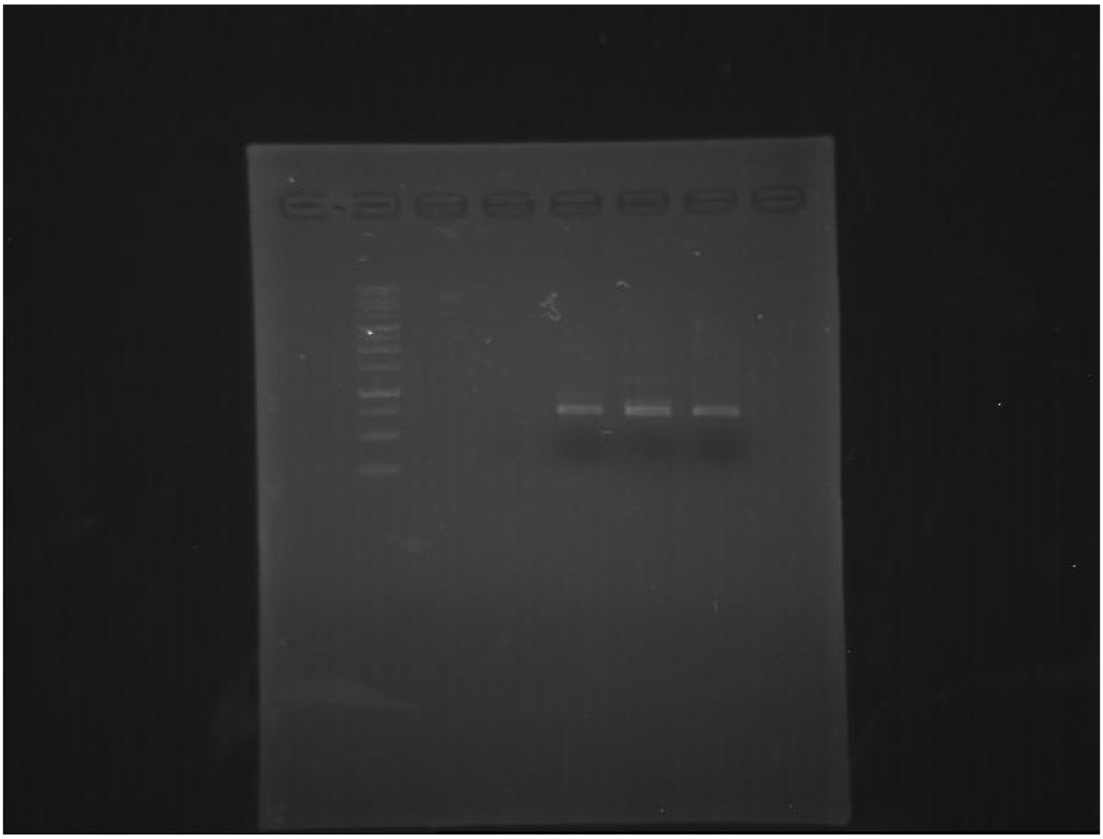

Fig.5b Left original figure

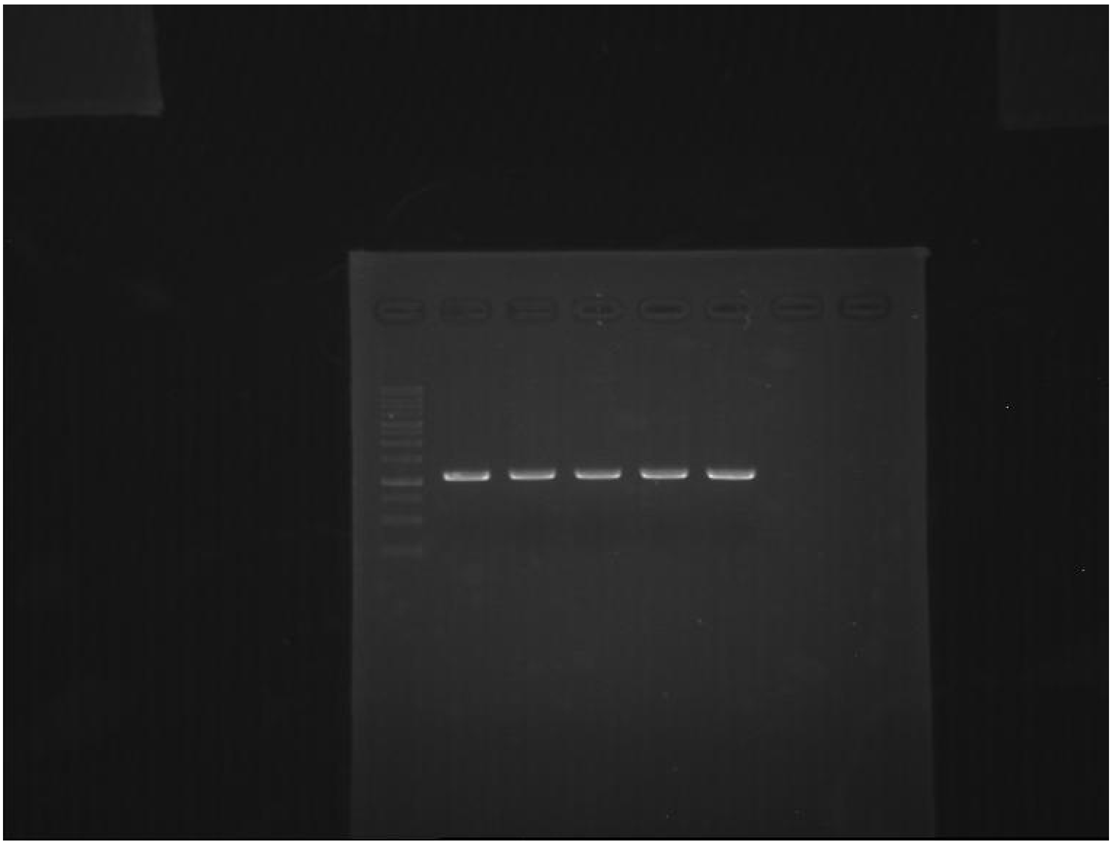

Fig.5b Right original figure

Fig.5(b). PCR analyses of *aph7”* transcripts in WT, apt single mutants, and KI mutants.
